# AI Chatbots vs. Traditional Sources: Dental Health Literacy and Confidence Among Dental Patients-A Cross-Sectional Study

**DOI:** 10.1016/j.identj.2026.109556

**Published:** 2026-04-20

**Authors:** Sachin Naik, Sajith Vellappally, Mohammed Alateek, Yasser Fahad Alrayyes, Abdul Aziz Abdullah Al Kheraif, Talal Mughaileth Alnassar, Gerhard Schmalz, Ziyad Mohammed Alsultan, Haya Alayadi, Nandita Suresh, Sukumaran Anil, Avneesh Chopra

**Affiliations:** a Dental Health Department, College of Applied Medical Sciences, King Saud University, Riyadh, Saudi Arabia; b Dental University Hospital, King Saud University, Riyadh, Saudi Arabia; c Prosthetic Dental Science Department, College of Dentistry, King Saud University, Riyadh, Saudi Arabia; d Department of Conservative Dentistry and Periodontology, Brandenburg Medical School Theodor Fontane (MHB), Brandenburg an der Havel, Germany; e Department of Oral and Maxillofacial Diseases, Helsinki University and University Hospital, Helsinki, Finland; f Global Research Cell, Dr. D. Y. Patil Dental College and Hospital, Dr. D. Y. Patil Vidyapeeth, Pune, India; g Faculty of Dentistry, Chulalongkorn University, Bangkok, Thailand; h International Faculty, Manipal School of Life Sciences, Manipal Academy of Higher Education, Manipal, Karnataka, India

**Keywords:** Artificial intelligence, Chatbots, eHealth literacy, Health information, Patient attitudes, Dental health

## Abstract

**Background and Objectives:**

Given the increasing use of artificial intelligence (AI) tools for dental health information, this study compared patients' perceived health information literacy for AI chatbots, measured using the Artificial Intelligence–eHealth Literacy Scale (AI-eHEALS), with traditional health information literacy, measured using the Traditional Health Information Literacy Scale (THILS) and examined how literacy and confidence influence information source preferences.

**Materials and Methods:**

A cross-sectional study was conducted at King Saud University Dental Hospital in Riyadh, Saudi Arabia, collecting responses from 474 adult dental patients using 8 validated items from AI-eHEALS and THILS. Domain-wise scores were calculated, and assumptions for parametric testing were verified. Group differences were analysed using independent t-tests. Structural equation modelling (SEM) examined pathways linking literacy domains, confidence, and information-source preferences, with perceived knowledge and evaluation items combined into a single AI-eHEALS literacy construct.

**Results:**

The THILS scores were significantly higher than those on the AI-eHEALS across all domains (*p* < .001). AI-eHEALS scores were positively associated with prior AI use, male gender, and employment status, while THILS showed few demographic links. SEM indicated that combined literacy (knowledge + evaluation) predicted confidence in using AI tools (β = 0.62). Usefulness items showed weak loadings and were excluded, improving model reliability. Participants reported moderate familiarity with AI chatbots; however, they demonstrated higher scores and greater agreement with traditional sources.

**Conclusions:**

The findings indicate that traditional dental health sources remain preferred over AI chatbots for health information, even with improved digital literacy. The study provides insight into patient perceptions of AI tools in dental care and suggests a need for enhanced AI literacy education and transparent communication about AI capabilities and limitations in health information contexts.

## Introduction

Digital health information delivery has transformed how patients seek, evaluate, and use medical knowledge. The widespread availability of online health platforms has democratized access to information; however, this proliferation creates challenges for patients in distinguishing reliable, evidence-based information from potentially misleading or inaccurate content. The ability to effectively navigate and appraise digital health information, a competency termed eHealth literacy, has emerged as an essential skill in the modern healthcare landscape.^1^ Digital health solutions are now widely used, reflecting a global shift toward online medical platforms. Artificial intelligence (AI) driven chatbots and large language models (LLMs) represent a major technological shift in patient engagement and access to health information. Today’s AI tools, unlike static websites or scripted chatbots, produce responses dynamically, word by word, based on patterns found in extensive data. They feel conversational and adaptive, yet their outputs remain uncertain. Because LLMs generate probabilistic rather than deterministic outputs, users must interpret information under inherent uncertainty, which may alter how credibility and trust are assessed.^2^

In dentistry, artificial intelligence is increasingly recognized as a transformative technology across clinical practice, education, and research.^3^^,^^4^ A recent review discussed AI fundamentals and contemporary applications in dentistry, emphasizing the breadth of AI integration across diagnostic, therapeutic, and communicative domains.^5^^,^^6^ Additionally, structured reporting guidelines for AI research in dentistry have been proposed to enhance methodological rigor and transparency.^5^ Dental educators have acknowledged AI’s growing value in knowledge acquisition (74.3%), clinical decision-making (63.6%), and research (68.5%).^3^ However, educator-level adoption does not necessarily translate to patient-level confidence or preference.

As AI rapidly enters dental care and communication, it remains unclear whether patients will trust AI-generated information over traditional dental health sources such as dentists, pamphlets, or clinic websites. This uncertainty supports conceptualizing AI literacy as a distinct extension of health literacy, encompassing the ability to interpret machine-generated outputs, assess credibility under uncertainty, and calibrate trust appropriately.^7^ Ensuring accessible dental health information is crucial, given that over 3.5 billion people worldwide suffer from oral diseases, including a high prevalence of dental caries and periodontal conditions requiring treatment. Building adequate oral health literacy is equally important so that people can interpret complex preventive and treatment information and apply it appropriately. Patients require evidence-based guidance to navigate and identify reliable, scientifically supported information within this complex information environment.^8^^,^^9^

Evidence suggests that AI chatbots have the potential to enhance the delivery of dental knowledge, provide tailored and scalable advice, and offer cost-effective support, which may be particularly beneficial for populations with limited access to professional dental care.^10^ However, these benefits should be interpreted cautiously, as the evidence base remains limited and context-dependent. Evidence suggests that when AI technologies are used for health information delivery, they introduce unique interpretive challenges that go beyond traditional health and eHealth literacy, requiring users to navigate algorithmic behavior, output validity, and contextual trust calibration.^11^ Although promising for information delivery, AI chatbots face significant limitations. These systems may produce inaccurate or outdated information, lack accountability mechanisms, and be unable to replicate the nuanced clinical judgment of experienced professionals. Furthermore, the absence of professional licensing, quality control standards, and transparent algorithms raises concerns about reliability and patient safety. Despite potential benefits, concerns remain about accuracy, hallucinations, performance variability, lack of regulation, and data privacy risks. These limitations reinforce the need for empirical evaluation at the patient level.^5^^,^^12^^,^^13^ In clinical settings, AI-generated recommendations must be verified against current evidence and adapted to individual patient contexts.

Clinicians, academic websites, and professional health organizations represent established, regulated sources of dental health information. These traditional sources benefit from peer review, professional accountability, and alignment with current clinical guidelines. Dental professionals maintain ethical obligations and legal liability for health recommendations, creating inherent incentives for accuracy and evidence-based practice. Clinicians, academic websites, professional platforms, and peer-reviewed literature provide individualized assessment, accountability, and evidence, but are less accessible, less interactive, and less affordable than unverified online content.^14^ However, accessibility barriers, including geographic limitations, cost, language preferences, and availability, may restrict patient access to traditional sources, particularly in underserved populations.

Understanding how patients perceive and utilize AI-generated health information requires a multifaceted theoretical lens. The eHealth Literacy Model proposed by Norman and Skinner^15^ conceptualizes health literacy as encompassing the ability to seek, find, understand, and appraise health information from electronic sources, providing the foundational framework for the present study’s measurement approach. The Technology Acceptance Model (TAM) posits that perceived usefulness and perceived ease of use are primary determinants of technology adoption, offering a framework for understanding why patients may prefer traditional over AI-based sources.^16^ Additionally, theories of health information-seeking behavior suggest that source credibility, accessibility, and prior experience shape individuals’ information preferences.^17^ Trust in digital health technologies has emerged as a critical mediator between digital literacy and sustained technology engagement, particularly for AI-based tools where algorithmic transparency remains limited.^2^

Emerging literature suggests that the unique characteristics of generative AI systems, including algorithmic opacity, probabilistic output generation, and contextual variability, require users to develop competencies beyond traditional eHealth literacy, often conceptualized as AI literacy.^18^^,^^19^ Recent scholarship defines AI literacy as distinct from general eHealth literacy, encompassing competencies in interpreting machine-generated outputs, evaluating algorithmic reliability, and calibrating trust under uncertainty.^20^ Traditional eHealth literacy frameworks emphasize skills such as locating, understanding, and appraising online health information, yet AI-driven tools introduce additional challenges, including opaque algorithms, probabilistic outputs, and the need to critically interpret machine-generated content.^21^^,^^22^ Systematic reviews indicate that integrating AI into health education and communication reshapes informational demands and highlights gaps in traditional literacy models, notably in adaptive content delivery, ethical concerns, and interpretive complexity.^23^

Importantly, AI literacy is conceptually distinct from general eHealth or digital literacy. While eHealth literacy broadly addresses the ability to seek, find, understand, and appraise health information from electronic sources, AI literacy encompasses additional competencies specific to AI-generated content. These include understanding how AI systems generate outputs, evaluating the reliability of algorithmically produced information, recognizing the potential for AI hallucinations and biases, and critically appraising the applicability of AI-generated recommendations to individual health contexts.^24^^,^^25^ Rather than replacing traditional eHealth literacy, AI literacy can be conceptualized as an advanced layer that incorporates competencies related to probabilistic reasoning, trust calibration, and evaluation of generative outputs. The AI-eHEALS instrument used in this study was designed to capture these AI-specific evaluative competencies rather than general digital navigation skills.

The eHealth Literacy Scale (eHEALS), developed by Norman and Skinner, is a validated self-report instrument widely used to assess perceived skills in finding, evaluating, and applying electronic health information.^15^ In this study, we adapted eHEALS to create 2 parallel instruments: the Artificial Intelligence-eHealth Literacy Scale (AI-eHEALS) for assessing literacy related to AI chatbot-generated information, and the Traditional Health Information Literacy Scale (THILS) for assessing literacy related to conventional sources. The integration of AI into health communication raises important regulatory considerations. The absence of established licensing frameworks, professional accountability mechanisms, and standardized quality benchmarks for AI-generated health information creates uncertainty for both patients and providers.^12^^,^^13^ Understanding patient perceptions in this evolving regulatory landscape is essential for informing evidence-based policy development.

While patients express preferences for different health information sources, empirical evidence directly comparing AI-mediated dental information with traditional sources at the patient level remains limited. Specifically, little is known about how the modality of information influences perceived literacy, confidence in decision-making, and subsequent source preference. This gap is critical as LLMs are increasingly embedded within consumer health platforms and dental communication systems.^7^ The present study addresses this evidence gap by conducting a comprehensive comparative analysis of patients' perspectives on AI chatbots and traditional sources of dental health information. Grounded in health literacy theory and information processing models, this study proposes that information modality influences perceived literacy, which in turn shapes decision-making confidence and source preference. Within this framework, perceived literacy functions as a cognitive mediator between information exposure and evaluative judgment. In this study, perceived literacy related to AI and traditional sources was measured using validated instruments designed to capture these distinct domains.

The primary aim was to evaluate how perceived literacy, decision-making confidence, and information source preferences differ when accessing information through AI chatbots versus conventional sources. Specifically, this study aimed to (1) assess and compare patient-reported eHealth literacy for AI-generated and traditional dental health information; (2) examine whether literacy predicts confidence in using these information sources; and (3) identify demographic and behavioral factors associated with literacy and confidence in each source type. It was hypothesized that patients would demonstrate modality-specific differences in literacy perception, decision confidence, and source preference, with these differences moderated by demographic characteristics and prior digital health experience. We further hypothesized that traditional sources would be preferred due to higher perceived reliability and professional accountability, and that higher AI literacy would correlate with greater confidence in AI-generated content.

## Materials and methods

### Study design and setting

This cross-sectional study was conducted at King Saud University Dental Hospital, Riyadh, Saudi Arabia, from January to April 2025. The hospital functions as a tertiary referral centre for dental care and a teaching facility, with an annual patient population exceeding 15,000. The study adhered to the Strengthening the Reporting of Observational Studies in Epidemiology (STROBE) guidelines for reporting cross-sectional observational research, thereby enhancing transparency and reproducibility.^26^

### Participant recruitment

Eligible participants were identified through the hospital’s patient management system. Inclusion criteria were: (1) age 18–75 years; (2) attendance for routine dental examination (non-emergency); (3) ability to read and understand written Arabic or English; and (4) absence of cognitive impairment. Exclusion criteria were previous participation in the study and unwillingness to provide informed consent. Recruitment followed a systematic procedure designed to minimize selection bias and ensure demographic diversity. Patients were invited via secure digital platforms, including email and WhatsApp. Participants completed an electronic questionnaire using Microsoft Forms, with all items set as mandatory to prevent incomplete responses. Following the Dillman Tailored Design Method, recruitment involved 5 sequential steps: pre-notification, invitation with the questionnaire, a reminder email after one week, a follow-up link at 2 weeks, and a final WhatsApp message after 3 weeks. All communications featured customized greetings, institutional branding, and researcher contact details. Of 1,000 patients invited, 474 completed the survey, yielding a response rate of 47.4%.

### Sample size determination

An a priori power analysis conducted using G*Power (α = 0.05, statistical power = 0.95) indicated a minimum of 138 participants for multiple regression with 5 predictors, assuming a medium effect size (f² = 0.15). However, the planned structural equation model (SEM) involved 32 estimated parameters, which required a substantially larger sample size. Following established SEM guidelines recommending 10–20 observations per estimated parameter, a minimum of 474 participants was targeted. The achieved sample of 474 respondents provided an observation-to-parameter ratio of 14.8:1, satisfying recommended thresholds for stable parameter estimation.

### Measures

#### AI-eHEALS and THILS

The study employed an adaptation of the eHealth Literacy Scale (eHEALS), a validated self-report instrument measuring perceived skills in finding, evaluating, and applying electronic health information.^15^ Two parallel scales were developed: the Artificial Intelligence–eHealth Literacy Scale (AI-eHEALS) and the Traditional Health Information Literacy Scale (THILS). The AI-eHEALS and THILS were developed by retaining the original eHEALS item structure, domain framework, and response format, while modifying only the information-source referent from general online health resources to AI chatbots (AI-eHEALS) or to traditional sources such as clinician consultations, academic websites, and professional organizations (THILS). The core domains of perceived knowledge and awareness, evaluation skills, confidence in application, and perceived usefulness were preserved to maintain conceptual equivalence between versions. In this study, AI-eHEALS assesses participants’ perceived competence in seeking, evaluating, and applying AI-generated health information, including from AI chatbots, rather than their technical ability to operate these systems. This approach is consistent with established health literacy frameworks that conceptualize literacy as an evaluative and decision-making competence rather than operational proficiency.^24^^,^^25^

The domains were assessed through paired items (Q1a–Q8a for AI-eHEALS and Q1b–Q8b for THILS), allowing direct comparison between AI-based and traditional sources (Table 1). Knowledge items assessed perceived ability to locate and access health information (e.g., “I can use AI chatbots to obtain health information”). Evaluation items assessed critical appraisal skills (e.g., “I can critically appraise the reliability of health information generated by AI chatbots”). Confidence items assessed perceived ability to apply information to health decisions. All items were scored on a 5-point Likert scale (1 = Strongly Disagree to 5 = Strongly Agree). Supplementary Table 1 provides the complete item inventory and response options.

**Table 1:** Domains and Items of AI-eHEALS and THILS

Domain	Items (AI-eHEALS)	Items (THILS)
Knowledge	Q1a–Q2a	Q1b–Q2b
Evaluation skills	Q3a, Q7a	Q3b, Q7b
Confidence	Q4a, Q6a, Q8a	Q4b, Q6b, Q8b
Usefulness	Q5a	Q5b

Participants were not directed to evaluate any specific AI chatbot platform. Instead, the survey instructions broadly referenced ‘AI chatbots used for health information,’ allowing participants to respond based on their individual exposure to and experience with AI-based conversational tools, including but not limited to ChatGPT, Google Gemini, and similar platforms. This approach reflects the heterogeneity of consumer use of AI tools and is consistent with the study’s aim to capture general perceptions rather than platform-specific evaluations.

#### Translation and validation

The Arabic version of the instrument underwent forward–backward translation by independent bilingual experts, followed by reconciliation, cognitive interviews, and pilot testing (n = 50). Pilot participants provided feedback on item clarity, wording, and relevance. Feedback was reviewed by the research team, and minor revisions were made to ensure linguistic and cultural appropriateness for dental patients in Saudi Arabia. Content validity was assessed by a panel of dental health and digital health experts, and face validity was established through pilot testing. Internal consistency was high for both subscales (Cronbach’s α = 0.876 for AI-eHEALS; α = 0.853 for THILS). The development of AI-eHEALS represents a systematic adaptation of the validated eHEALS instrument rather than de novo instrument development. Construct equivalence between AI-eHEALS and THILS is supported by the parallel item structure, comparable internal consistency coefficients, and the 2-factor EFA solution that clearly distinguished AI-based from traditional items.

#### Confidence, usefulness, and demographics

Two additional items measured confidence and perceived usefulness: “I feel confident making health decisions based on AI chatbot information” (confidence) and “I find AI chatbots useful for accessing health information” (usefulness), measured on the same 5-point Likert scale. Demographic variables included age (in years), gender (male/female), education level, employment status (employed/unemployed), and prior use of AI chatbots for health information (yes/no/unsure). Prior chatbot use was dichotomized for analysis (yes vs. no/unsure).

### Data collection and management

Participants completed the electronic questionnaire via Microsoft Forms. Surveys were available in both Arabic and English to accommodate language preferences. All items were set as mandatory to prevent incomplete submissions. Data were exported to a secure, password-protected database and de-identified for analysis. All responses were kept confidential, with only authorized study investigators having access to identifiable information.

### Statistical analysis

#### Preliminary analyses

Data analysis was performed using SPSS version 29 and AMOS version 29. Domain-level composite scores were calculated as the arithmetic mean of constituent items within each domain for both AI-eHEALS and THILS. Item allocation to each domain was guided by the theoretical framework of eHEALS^15^ and subsequent empirical studies,^27^ reflecting the original conceptual domain structure rather than the final SEM specification. Descriptive statistics were computed for all variables. Assumptions for parametric testing, including normality (assessed using Shapiro–Wilk tests and skewness–kurtosis indices), homoscedasticity (Levene’s test and residual plots), and the absence of influential outliers (leverage statistics), were verified prior to inferential analyses. No transformations were necessary.

#### Group comparisons

Item-level differences between AI-eHEALS and THILS were examined using paired-sample t-tests. Domain-level composite scores were also generated and plotted to compare score profiles across sources. Statistical significance was set at *p* < .05 (2-tailed). Effect sizes (Cohen’s d) were calculated for all comparisons.

#### Multiple linear regression analysis

Two multiple linear regression models were estimated to identify predictors of AI-eHEALS and THILS composite scores. For each model, a composite mean score was computed as the dependent variable. Predictors included age, gender, education level, employment status, and prior AI chatbot use. Education level was dummy-coded with “illiterate” as the reference category, and employment status with “unemployed” as the reference category. Model statistics (R, R², F-tests) and 95% confidence intervals were reported. Domain-level composites were retained for regression analyses despite combining knowledge and evaluation skills into a single latent factor for SEM, as descriptive and regression analyses do not impose latent structural assumptions and enable comparability with prior eHEALS studies.

#### Factor structure analysis: information source differentiation

Exploratory factor analysis (EFA) was conducted on all questionnaire items to examine whether participants’ responses differentiated between AI-based and traditional information sources. Principal axis factoring with oblimin (oblique) rotation was employed. Factor retention was determined using multiple predefined criteria: eigenvalues greater than 1.0 (Kaiser criterion), inspection of the scree plot, parallel analysis, and interpretability of the factor solution. Items were retained if primary loadings were ≥0.50; cross-loadings ≥0.40 were considered meaningful, and loading differences <0.20 between factors were evaluated as potential conceptual overlap.

This analysis supported a 2-factor solution broadly aligned with AI-eHEALS and THILS, jointly explaining 46.71% of the total variance (Factor 1 = 30.31%; Factor 2 = 16.40%). This proportion of variance explained is comparable to values reported in prior eHealth literacy validation studies across multidimensional health literacy instruments. Sampling adequacy was verified via the Kaiser–Meyer–Olkin measure and Bartlett’s test of sphericity (Supplementary Table 2). Detailed factor loadings, cross-loadings, and communalities are reported in Supplementary Tables 3 and 4.

#### Latent construct specification for SEM

A separate EFA was conducted on the combined item set to examine the dimensionality of literacy constructs, independent of the information-source context. This analysis addressed a distinct methodological objective from the source-differentiation EFA described above: whereas the first EFA examined whether participants’ responses differentiated between AI-based and traditional information sources, this second EFA examined the internal dimensionality of literacy constructs within each source category. These analyses were conducted sequentially to avoid analytical redundancy.

Although the conceptual framework initially distinguished knowledge and evaluation skills as separate domains, EFA results revealed substantial empirical overlap between them. Items intended to represent both domains loaded predominantly on a dominant general factor, with several items exhibiting meaningful cross-loadings ≥0.40 under oblique rotation (Supplementary Table 3), indicating weak discriminant validity between the proposed subdomains. The first extracted factor accounted for the largest proportion of shared variance (Eigenvalue = 4.85; 30.31%), with a second factor contributing additional but less distinct variance (Eigenvalue = 2.62; cumulative variance = 46.71%), suggesting a moderate underlying literacy construct rather than clearly separable knowledge and evaluation dimensions.

To assess discriminant validity between knowledge and evaluation factors, inter-factor correlations from the oblique rotation were examined. The correlation between these factors exceeded 0.70, indicating substantial empirical overlap that warranted consolidation. Additionally, average variance extracted (AVE) and composite reliability (CR) values were computed; although convergent validity was adequate, the high inter-factor correlations indicated insufficient discriminant validity to justify treating the constructs as separate.

The decision to consolidate was further supported by comparative model testing. The separate domain model demonstrated poor fit (χ²/df = 32.99), and the standardized latent correlation between knowledge and evaluation was extremely high (r = 0.961), confirming lack of discriminant validity and substantial construct redundancy. In contrast, the merged single-factor literacy model demonstrated markedly improved fit (CFI = 0.971; TLI = 0.954; χ²/df = 4.50; RMSEA = 0.086), supporting consolidation. Conceptually, knowledge acquisition and evaluative capacity represent interdependent components of a unified literacy process—namely, the ability to access, interpret, and critically appraise health information. Based on convergent evidence from EFA results, SEM diagnostics, and theoretical coherence, items from both domains were therefore merged into a single latent literacy construct for SEM analysis.

#### SEM path analysis

Structural equation modelling (SEM) was conducted to examine the pathways linking literacy, confidence, and information source preference. Separate models were specified for AI-eHEALS and THILS, with literacy (consolidated knowledge + evaluation) as a latent predictor of confidence. For the AI-eHEALS model, the literacy latent construct was specified using items Q1a, Q2a, Q3a, and Q7a, while confidence in applying information was specified using items Q4a, Q6a, and Q8a. Maximum likelihood estimation was used.

Confirmatory factor analysis (CFA) demonstrated strong standardized factor loadings for the literacy construct (0.696–0.879) and the confidence construct (0.796–0.827), confirming satisfactory convergent validity and construct reliability. Model fit was evaluated using multiple indices: Comparative Fit Index (CFI ≥0.95), Tucker–Lewis Index (TLI), Root Mean Square Error of Approximation (RMSEA ≤0.08), and chi-square/degrees of freedom ratio (≤5.0). The final model demonstrated acceptable fit (CFI = 0.971; TLI = 0.954; χ²/df = 4.50; RMSEA = 0.086). The RMSEA value of 0.086 falls within the acceptable range but should be interpreted with appropriate caution, as values below 0.08 are generally considered indicative of good fit.^28^ The marginally elevated RMSEA likely reflects the complexity of the measurement model relative to the sample size, and this limitation should be considered when interpreting the structural paths.

Items intended to measure perceived usefulness exhibited weak factor loadings (λ = 0.22–0.35) and nontrivial cross-loadings, thereby failing to form a coherent latent construct and adversely affecting model fit. Consequently, the usefulness dimension was excluded from the SEM to preserve model reliability and validity, while being retained for descriptive and regression analyses to ensure comprehensive reporting of participant perceptions.

Demographic variables (age, gender, education level, employment status, prior chatbot use) were included as covariates in the model predicting literacy scores. Standardized path coefficients (β) and 95% confidence intervals were computed for all structural paths. Indirect effects (literacy → confidence) were estimated using bootstrapped standard errors (5,000 resamples).

### Ethical considerations

This study was approved by the Institutional Review Board (IRB) of King Saud University (Reference No. IRB E-24-9321). All participants provided written informed consent before completing the survey. The study adhered to the Declaration of Helsinki. All data were maintained in secure, password-protected databases accessible only to authorized study personnel. Participant privacy and autonomy were protected throughout the research process.

## Results

### Participant characteristics

A total of 474 dental patients participated in the study. Table 2 presents demographic characteristics. The mean age was 37.2 years (SD = 14.8, range: 18-72 years). The sample was 58.4% female (n = 277) and 41.6% male (n = 197). Regarding employment, 62.0% (n = 294) were employed, and 38.0% (n = 180) were unemployed (including homemakers and students). Prior experience with AI chatbots for health information was reported by 48.1% (n = 228), 34.2% (n = 162) reported no experience, and 17.7% (n = 84) were unsure.

**Table 2:** Participant demographic characteristics (n = 474)

Variable	Category	n	Percentage (%)
Age (years)	Mean ± SD: 37.2 ± 14.8	Range: 18–72	
Gender	Female	277	58.4
	Male	197	41.6
Employment	Employed	294	62.0
	Unemployed	180	38.0
Prior AI Chatbot use	Yes	228	48.1
	No	162	34.2
	Unsure	84	17.7

### Descriptive statistics for AI-eHEALS and THILS

**Fig. 1:**
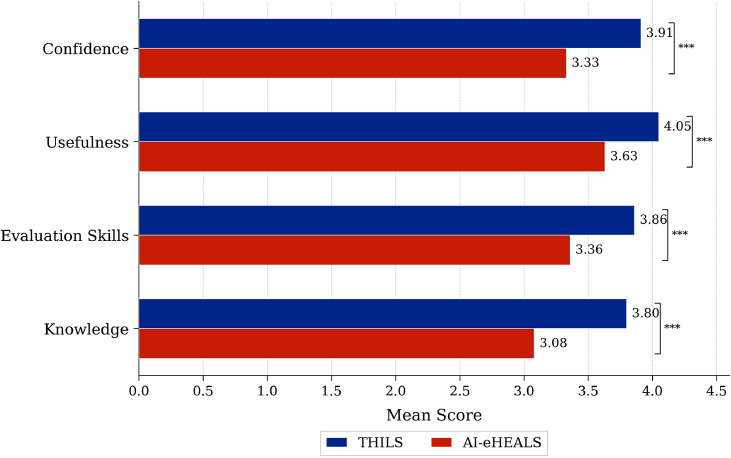
Domain-wise mean scores for AI-eHEALS and THILS across 4 literacy domains (Knowledge, Evaluation Skills, Usefulness, and Confidence). THILS scores (blue) were significantly higher than AI-eHEALS scores (red) across all domains (*p* < .001, paired-sample t-tests). Values represent mean scores on a 5-point Likert scale (1 = Strongly disagree to 5 = Strongly agree). AI-eHEALS = Artificial Intelligence–eHealth Literacy Scale; THILS = Traditional Health Information Literacy Scale. ***p < .001.

Table 3 presents mean domain scores for both instruments. THILS knowledge scores (M = 4.21, SD = 0.68) were significantly higher than AI-eHEALS knowledge scores (M = 2.87, SD = 1.12), t(472) = −16.84, *p* < .001, Cohen's d = −1.54. THILS evaluation scores (M = 4.08, SD = 0.74) also exceeded AI-eHEALS evaluation scores (M = 2.76, SD = 1.18), t(472) = −15.62, *p* < .001, Cohen's d = −1.43. Confidence in AI tools (M = 2.91, SD = 1.24) was lower than confidence in traditional sources (M = 4.32, SD = 0.71), t(472) = −17.23, *p* < .001, Cohen's d = −1.58 (Figure 1). Higher mean scores and agreement rates were observed for traditional sources compared with AI chatbots across all measured items.

**Table 3:** Descriptive statistics for AI-eHEALS and THILS domain scores

Scale & domain	M	SD	Range	Cronbach's α
AI-eHEALS knowledge	2.87	1.12	1–5	0.81
AI-eHEALS evaluation	2.76	1.18	1–5	0.84
AI-eHEALS total	2.81	1.08	1–5	0.876
THILS knowledge	4.21	0.68	1–5	0.78
THILS evaluation	4.08	0.74	1–5	0.81
THILS total	4.15	0.67	1–5	0.853

### Factor structure analysis

Exploratory Factor Analysis (EFA) examined whether items differentiated between AI-based and traditional sources. A 2-factor solution emerged, with the first factor comprising all AI-eHEALS items (eigenvalue = 4.12) and the second factor comprising all THILS items (eigenvalue = 3.84). This pattern confirmed source-based differentiation, explaining 46.71% of total variance. All primary factor loadings exceeded 0.62, and cross-loadings were minimal (max = 0.18).

A second EFA, conducted separately within the AI-eHEALS and THILS, tested whether knowledge and evaluation items formed distinct factors. Inter-factor correlations exceeded 0.70 for both scales, and comparative model testing confirmed that a single consolidated literacy factor provided superior fit compared with a 2-factor specification. Accordingly, knowledge and evaluation items were combined into a unified literacy construct for subsequent SEM analyses.

### Demographic associations with AI-eHEALS and THILS scores

Table 4 presents results from multiple regression analyses predicting AI-eHEALS and THILS literacy scores. For AI-eHEALS, prior chatbot use was a significant positive predictor (B = 0.48, 95% CI [0.21, 0.75], *p* = .001), indicating a positive association between prior chatbot use and AI perception scores. Male gender was associated with higher AI-eHEALS scores (B = 0.32, 95% CI [0.08, 0.56], *p* = .009). Age was a significant negative predictor (B = −0.021, 95% CI [−0.032, −0.010], *p* < .001), indicating a negative association between age and AI perception scores. Employment was a significant predictor (B = 0.28, 95% CI [0.06, 0.50], *p* = .014), with employed individuals scoring higher than unemployed participants.

**Table 4:** Regression analysis: demographic predictors of literacy scores

Predictor	AI-eHEALS B (95% CI)	*p*-value	THILS B (95% CI)	*p*-value
Age (per year)	−0.021 (−0.032, −0.010)	<.001	−0.008 (−0.016, 0.001)	.068
Male gender	0.32 (0.08, 0.56)	.009	0.18 (−0.01, 0.37)	.048
Employed	0.28 (0.06, 0.50)	.014	0.12 (−0.08, 0.32)	.232
Prior Chatbot Use	0.48 (0.21, 0.75)	.001	0.21 (−0.01, 0.43)	.062

For THILS scores, age and employment were non-significant predictors (*p* > .05). Gender was associated with THILS scores (B = 0.18, *p* = .048), and prior chatbot use showed a marginally significant positive association (B = 0.21, *p* = .062).

### Structural equation modelling results

**Fig. 2:**
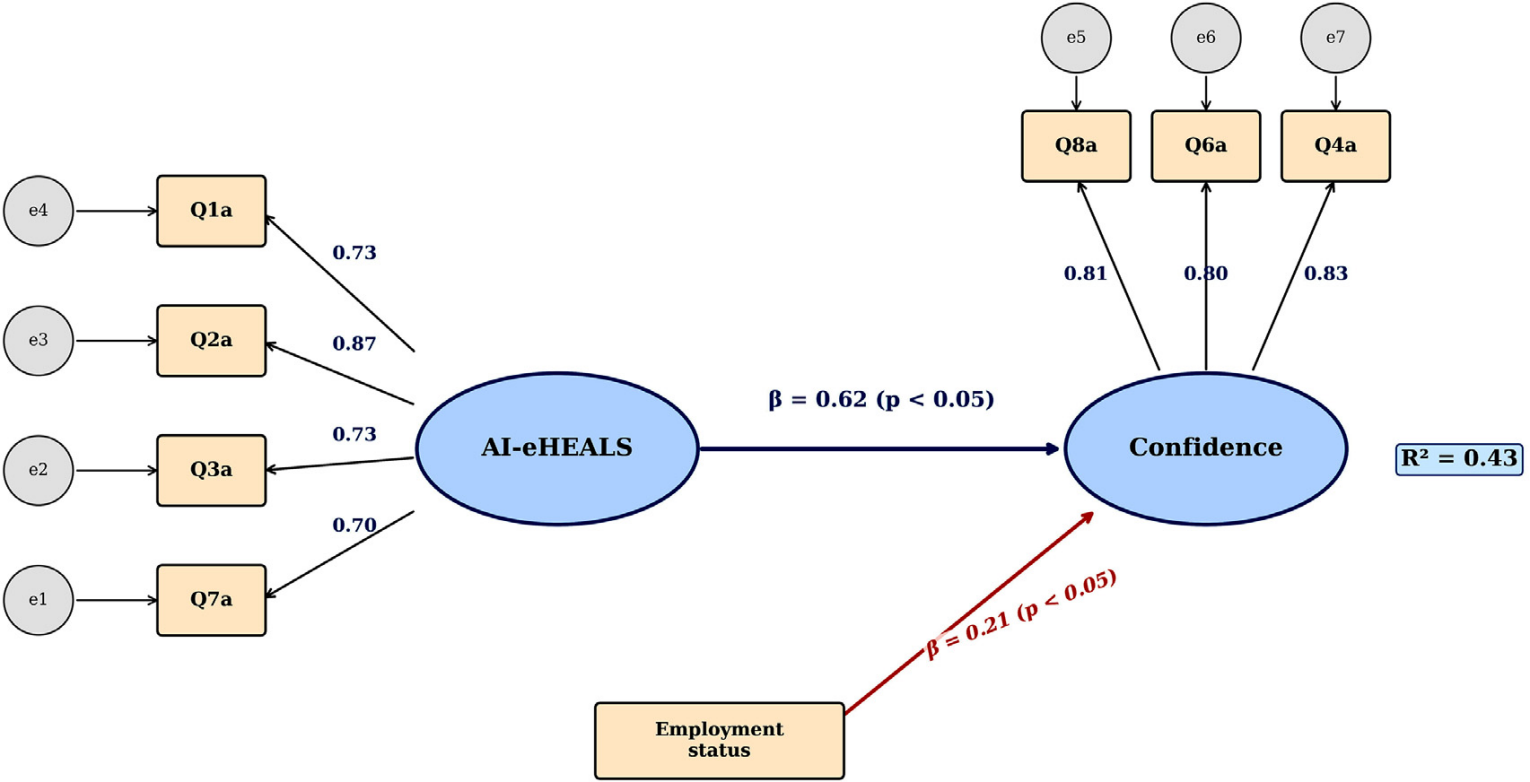
Structural equation model illustrating the relationships between AI-eHEALS literacy, employment status, and confidence in using AI-based dental health information. Ellipses represent latent constructs; rectangles represent observed indicator variables; circles represent error terms (e1-e7). Standardized factor loadings are displayed on paths from latent constructs to their respective indicators. The primary structural path from AI-eHEALS literacy to Confidence was statistically significant (β = 0.62, *p* < .05), as was the path from Employment status to Confidence (β = 0.21, *p* < .05). The model explained 43% of the variance in Confidence (R² = 0.43). AI-eHEALS = Artificial Intelligence–eHealth Literacy Scale (items Q1a, Q2a, Q3a, Q7a); Confidence construct measured by items Q4a, Q6a, Q8a.

Separate SEM models examined pathways from literacy to confidence for AI-eHEALS and THILS. For AI-eHEALS (Table 5), the model demonstrated adequate fit (CFI = 0.971, RMSEA = 0.086, χ²/df = 4.50). The pathway from AI literacy to confidence was strong and statistically significant (β = 0.62, 95% CI [0.54, 0.70], *p* < .001). Higher perceived literacy was positively associated with confidence in using AI tools. Among demographic covariates, prior chatbot use was associated with higher literacy scores (β = 0.31, *p* < .001), and male gender predicted higher literacy (β = 0.15, *p* = .008). Age negatively predicted AI-eHEALS scores (β = −0.18, *p* < .001), and employment status was a significant predictor (β = 0.13, *p* = .022) (Figure 2).

**Table 5:** SEM path coefficients for AI-eHEALS and THILS models

Pathway	β (95% CI)	SE	*p*-value	Sig.
**AI-eHEALS Model**				
Literacy → Confidence	0.62 (0.54, 0.70)	0.041	<.001	⁎⁎⁎
Prior Use → Literacy	0.31 (0.17, 0.45)	0.072	<.001	⁎⁎⁎
Male → Literacy	0.15 (0.04, 0.26)	0.058	.008	⁎⁎
Age → Literacy	−0.18 (−0.27, −0.09)	0.047	<.001	⁎⁎⁎
Employment → Literacy	0.13 (0.02, 0.24)	0.056	.022	*
**THILS model**				
Literacy → Confidence	0.71 (0.64, 0.78)	0.036	<.001	⁎⁎⁎
Male → Literacy	0.12 (−0.002, 0.24)	0.061	.053	†

Note. SE = Standard Error;

† *p* <.10;

⁎ *p* < .05;

⁎⁎ *p* < .01;

⁎⁎⁎ *p* < .001

For THILS (Table 5), the model also demonstrated acceptable fit (CFI = 0.968, RMSEA = 0.089, χ²/df = 4.72). The pathway from traditional literacy to confidence in traditional sources was strong (β = 0.71, 95% CI [0.64, 0.78], *p* < 0.001), with higher literacy related to traditional sources positively associated with confidence. Demographic predictors of THILS literacy were less pronounced; only gender approached significance (β = 0.12, *p* = 0.053).

### Model fit and validity

Both SEM models demonstrated acceptable overall fit. The AI-eHEALS model showed strong convergence (CFI = 0.971, TLI = 0.958) with an RMSEA of 0.086 and SRMR of 0.052. The THILS model showed similar adequacy (CFI = 0.968, TLI = 0.954, RMSEA = 0.089, SRMR = 0.056). These indices suggest that the theorized pathways from literacy to confidence are well supported by the data from both information sources (Figure 2).

### Item-level responses and patterns

Table 6 presents the percentage agreement (“Agree” + “Strongly Agree”) for each measured item. For AI-eHEALS items, agreement ranged from 31.4% to 42.8%, whereas THILS items showed agreement ranging from 82.3% to 91.6%. These data further illustrate the pervasive preference for and trust in traditional sources compared with AI chatbots (Figure 1).

**Table 6:** Item-level agreement rates

Item	% Agree (AI-eHEALS)	% Agree (THILS)
I can find health information...	36.9	85.4
I feel confident using [source] to find useful health information	38.2	87.1
I can critically appraise the reliability...	31.4	82.3
I can determine whether information is credible	35.6	88.9
I feel confident making health decisions based on [source]	39.7	91.6
Overall confidence in [source]	42.8	89.2

## Discussion

These findings align with, extend, and challenge existing evidence in the intersection of eHealth literacy, technology adoption, and patient preferences for dental health information. When interpreted through the theoretical frameworks outlined in the Introduction, the eHealth Literacy Model, the Technology Acceptance Model, and health information-seeking behavior theory, the observed patterns reveal meaningful insights about the determinants of patient engagement with AI-based dental health information.^15^^,^^16^ A key finding is the robust preference for traditional sources over AI chatbots across all measured dimensions. THILS scores substantially exceeded AI-eHEALS scores (Cohen's d range: −1.43 to −1.54), and agreement rates for traditional sources (82%–92%) were approximately double those for AI chatbots (31%-43%). These patterns reflect structural differences between the 2 information modalities. Traditional sources, particularly dental consultations and regulated professional organizations, operate within established accountability frameworks, licensing requirements, and professional ethics standards that create inherent quality-assurance mechanisms.^29^^,^^30^ In contrast, AI chatbots lack professional licensure, face limited regulatory oversight, and operate through opaque algorithmic processes that patients may not understand or trust.^4^^,^^31^

The cultural context of the study setting may also contribute to these findings. In Saudi Arabia, healthcare delivery is predominantly institution-based, and patients traditionally rely on direct consultation with dental professionals. This cultural orientation toward authority-based information sources may amplify the preference for traditional over AI-generated content, a pattern that may differ in healthcare systems with greater emphasis on patient self-management or in populations with higher baseline digital literacy. The strong pathway from literacy to confidence, particularly pronounced for traditional sources (β = 0.71), aligns with the eHealth Literacy Model's premise that competence in evaluating and applying health information drives confidence and engagement.^15^^,^^32^ These findings are consistent with the Technology Acceptance Model, which posits that perceived competence (analogous to perceived ease of use) facilitates technology adoption.^16^ The strong pathway from literacy to confidence observed here suggests that literacy is a proximal determinant of the self-efficacy required for sustained engagement with AI health tools.^33^

Demographic associations revealed important patterns. Prior exposure to AI chatbots strongly predicted higher AI literacy scores (β = 0.31), suggesting that familiarity builds perceived competence.^34^ This aligns with Technology Acceptance Model predictions that prior experience enhances perceived ease of use.^34^ Notably, age was a significant negative predictor of AI literacy (β = −0.18), consistent with broader literature documenting age-related differences in technology adoption and comfort.^35^^,^^36^ Male gender and employment status also predicted higher AI-eHEALS scores, suggesting that socioeconomic and gender factors may influence technology adoption.^37^^,^^38^ In contrast, demographic variables showed minimal association with THILS scores, indicating that traditional sources are perceived as accessible across demographic subgroups.^39^

The exclusion of the usefulness construct from the final SEM model warrants careful consideration. In the Technology Acceptance Model, perceived usefulness is a central predictor of technology adoption and sustained use.^34^ The weak factor loadings observed for usefulness items in this study may reflect the nascent stage of AI chatbot adoption among dental patients, in which perceptions of usefulness are unstable and heavily influenced by limited or inconsistent exposure.^40^ This exclusion may limit the model's ability to capture the full spectrum of factors driving AI adoption in healthcare. Future research should develop more psychometrically robust measures of perceived usefulness specific to AI health information tools, potentially incorporating dimensions such as perceived accuracy, relevance, and actionability of AI-generated content.^31^

These results have important implications for health policy and clinical practice. As AI tools proliferate in healthcare delivery, systems designers and policymakers should prioritize transparent communication about AI capabilities and limitations.^4^^,^^41^ Patients must understand when AI-generated information has been validated against clinical evidence, what safeguards are in place to prevent harmful errors, and how AI recommendations should be contextualized within their individual health circumstances. Additionally, efforts to enhance AI literacy, particularly targeting older adults and those with limited prior exposure to technology, may increase the appropriate use of AI health tools while maintaining critical appraisal skills to identify and reject unreliable algorithmic outputs.^32^^,^^35^

The findings further underscore the importance of maintaining and strengthening traditional health information sources, particularly in underserved populations where access to AI may be limited.^33^^,^^36^ While AI chatbots offer potential scalability advantages, the current study suggests that patients continue to prioritize vetted, professionally accountable sources when available. Optimal health information delivery may require an integrated approach combining AI's scalability with traditional sources' accountability, rather than positioning these modalities as competitors.^3^

## Limitations

Several limitations warrant consideration. First, the sample comprised dental patients at a single tertiary care center in Saudi Arabia, which may not represent the broader population of dental patients or the public. The cross-sectional design precludes causal inference; longitudinal research is needed to examine whether literacy changes over time or whether changes in literacy predict subsequent shifts in information source preferences.

Furthermore, the single-center recruitment strategy at King Saud University Dental Hospital in Riyadh, Saudi Arabia, limits the generalizability of the findings to other cultural, socioeconomic, and healthcare-system contexts. Patient perceptions of AI-generated health information may be influenced by factors such as national digital infrastructure, cultural attitudes toward technology and authority-based medicine, healthcare system organization, and the availability and quality of traditional information sources. Cross-cultural replication studies are needed to determine the extent to which these findings are generalizable.

The study relied on adapted instruments (AI-eHEALS and THILS) rather than previously published validated scales specific to AI health information literacy. While the adaptation process was systematic, and reliability coefficients were acceptable, longer validation studies with additional measurement approaches are needed to establish full psychometric equivalence. Additionally, the survey assessed general perceptions of 'AI chatbots' without directing participants to evaluate specific platforms; while this approach reflects heterogeneous real-world use, it may obscure platform-specific effects that could influence literacy and confidence assessments.

The use of self-reported measures introduces the possibility of social desirability bias, whereby participants may over-report confidence or competence to present themselves favourably. Finally, participant awareness that the study examined AI chatbots may have primed critical attitudes toward this information source relative to unprompted, naturalistic use patterns.

## Conclusion

This study provides empirical evidence that traditional dental health information sources remain substantially preferred over AI chatbots among dental patients in Saudi Arabia. The strong pathways from literacy to confidence for both information sources indicate that eHealth literacy is a key determinant of patient engagement and information source preference. Demographic factors, particularly age, gender, employment status, and prior chatbot exposure, predict AI literacy and adoption differently from traditional sources. As AI technologies continue to expand their role in healthcare delivery and health communication, policymakers and clinical educators must prioritize transparent communication about AI's capabilities and limitations. Efforts to enhance AI-specific literacy, distinct from general digital literacy, are essential to enabling patients to make informed decisions about relying on algorithmically generated health information. Simultaneously, strengthening traditional, professionally accountable sources remains critical for ensuring equitable access to reliable dental health information across all populations.

Future research should extend these findings through longitudinal designs, multicentre samples spanning diverse cultural and healthcare contexts, and more robust psychometric instruments specifically developed for AI health information literacy assessment. Understanding the evolving relationship between patients, AI tools, and traditional health information sources is essential for optimizing health communication in an era of rapid technological change.

## Patient consent statement

All participants provided written informed consent prior to study participation. Consent procedures were approved by the Institutional Review Board of King Saud University (Reference No. E-24-9321). Participants were informed of their right to withdraw from the study at any time without penalty, and all data were maintained confidentially throughout the research process.

## Author contributions

**Sachin Naik:** Conceptualization, Methodology, Formal analysis, Software, Validation, Writing – review & editing, Supervision. **Sajith Vellappally:** Conceptualization, Methodology, Project administration. **Mohammed Alateek:** Conceptualization, Methodology. **Avneesh Chopra:** Conceptualization, Methodology, Investigation, Writing – review & editing, Supervision. **Yasser Fahad Alrayyes:** Investigation, Data curation, Validation. **Abdul Aziz Abdullah Al Kheraif:** Investigation, Data curation, Resources. **Talal Mughaileth Alnassar:** Investigation, Data curation, Validation. **Gerhard Schmalz:** Investigation, Data curation, Validation. **Ziyad Mohammed Alsultan:** Writing – original draft, Visualization. **Haya Alayadi:** Writing – original draft, Visualization. **Nandita Suresh:** Writing – review & editing, Validation. **Sukumaran Anil:** Writing – review & editing, Supervision. All authors have materially participated in the research and/or article preparation, reviewed and approved the final version of the manuscript, and agreed to be accountable for all aspects of the work, ensuring its integrity and accuracy.

## Data availability statement

The datasets generated and analysed during this study are not publicly available due to participant confidentiality protections, but they are available from the corresponding authors upon reasonable request. Additional information is provided in the supplementary file.

## Conflict of interest

None disclosed.

## Appendix Supplementary materials

Image, application 1

## Footnotes

The supplementary material associated with this article can be found in the online version at the following doi:10.1016/j.identj.2026.109556.
