# Supplementary material for: AI Chatbots vs. Traditional Sources: Dental Health Literacy and Confidence Among Dental Patients-A Cross-Sectional Study
Source: Int Dent J. 2026 Apr 20;76(3):109556. doi: 10.1016/j.identj.2026.109556 (PMC13121421; doi:10.1016/j.identj.2026.109556)
Supplement: Supplementary file 1 [file mmc1.docx]

**Supplementary Material**

*AI Chatbots vs. Traditional Sources: Dental Health Literacy and Confidence Among Dental Patients—A Cross-Sectional Study*

*International Dental Journal*

**Supplementary Table 1.** Questionnaire items for AI-eHEALS and THILS.

| **Item** | **AI-eHEALS** | **Item** | **THILS** |
| --- | --- | --- | --- |
| Q1a | I know how to find dental health information using AI chatbots. | Q1b | I know how to find dental health information using traditional sources (e.g., brochures, dental consultations). |
| Q2a | I know what dental health resources are available through AI chatbots. | Q2b | I know where to find trustworthy traditional sources for dental health information. |
| Q3a | I can judge the quality of dental health information provided by AI chatbots. | Q3b | I can evaluate the reliability of dental health information from traditional sources. |
| Q4a | I feel confident applying dental health information obtained from AI chatbots to make decisions. | Q4b | I feel confident applying dental health information from traditional sources to make decisions. |
| Q5a | AI chatbots provide useful dental health information. | Q5b | Traditional sources provide useful dental health information. |
| Q6a | I am comfortable using AI chatbots to search for dental health information. | Q6b | I feel more comfortable using traditional sources for dental health information. |
| Q7a | I know how to get help if I have questions when using AI chatbots. | Q7b | I know how to get help if I have questions when using traditional sources. |
| Q8a | I feel confident using AI chatbots to make dental health decisions. | Q8b | I feel more confident using traditional sources to make dental health decisions. |

***Note:*** *AI-eHEALS = Artificial Intelligence–eHealth Literacy Scale; THILS = Traditional Health Information Literacy Scale. All items were scored on a 5-point Likert scale (1 = Strongly disagree to 5 = Strongly agree).*

**Supplementary Table 2.** Total variance explained by the two-factor EFA solution.

| **Factor** | **Eigenvalue** | **% Variance** | **Cumulative %** |
| --- | --- | --- | --- |
| 1 | 4.850 | 30.31 | 30.31 |
| 2 | 2.624 | 16.40 | 46.71 |

***Note:*** *Extraction method: Principal axis factoring. Only factors with eigenvalues > 1.0 are shown.*

**Supplementary Table 3.** Rotated factor matrix (two-factor solution with oblimin rotation).

| **Item** | **Factor 1 (AI-eHEALS)** | **Factor 2 (THILS)** |
| --- | --- | --- |
| Q2a | 0.655 | — |
| Q3a | 0.632 | — |
| Q6a | 0.632 | −0.416 |
| Q5a | 0.616 | — |
| Q7a | 0.602 | — |
| Q5b | 0.584 | 0.500 |
| Q4a | 0.555 | −0.453 |
| Q4b | 0.550 | — |
| Q2b | 0.532 | — |
| Q1a | 0.530 | — |
| Q3b | 0.530 | — |
| Q1b | 0.527 | — |
| Q8a | 0.499 | −0.479 |
| Q8b | 0.464 | 0.630 |
| Q6b | — | 0.561 |
| Q7b | 0.449 | 0.493 |

***Note:*** *Factor loadings < 0.40 are suppressed (—). Oblique rotation permits correlated factors; therefore, secondary loadings, including negative cross-loadings, are expected. The presence of cross-loadings ≥ 0.40 reflects overlapping literacy processes across information sources rather than model misspecification and supports subsequent construct consolidation in structural equation modelling.*

**Supplementary Table 4.** Communalities from exploratory factor analysis.

| **Item** | **Initial** | **Extraction** |
| --- | --- | --- |
| Q1a | 0.500 | 0.401 |
| Q1b | 0.393 | 0.341 |
| Q2a | 0.638 | 0.547 |
| Q2b | 0.411 | 0.365 |
| Q3a | 0.527 | 0.501 |
| Q3b | 0.409 | 0.389 |
| Q4a | 0.581 | 0.513 |
| Q4b | 0.403 | 0.407 |
| Q5a | 0.494 | 0.427 |
| Q5b | 0.574 | 0.591 |
| Q6a | 0.613 | 0.572 |
| Q6b | 0.451 | 0.454 |
| Q7a | 0.480 | 0.429 |
| Q7b | 0.451 | 0.493 |
| Q8a | 0.581 | 0.499 |
| Q8b | 0.553 | 0.613 |

***Note:*** *Extraction method: Principal axis factoring. Initial communalities reflect squared multiple correlations; extraction communalities indicate the proportion of each item’s variance accounted for by the retained factors.*
